# Equity and Efficiency of Health Resource Allocation of Chinese Medicine in Mainland China: 2013–2017

**DOI:** 10.3389/fpubh.2020.579269

**Published:** 2020-12-15

**Authors:** Zhengjun Li, Lili Yang, Shaoliang Tang, Yaoyao Bian

**Affiliations:** ^1^College of Health Economics Management, Nanjing University of Chinese Medicine, Nanjing, China; ^2^School of Management, University of St Andrews, St Andrews, United Kingdom; ^3^Jingwen Library, Nanjing University of Chinese Medicine, Nanjing, China; ^4^School of First Clinical Medicine, Nanjing University of Chinese Medicine, Nanjing, China; ^5^School of Nursing, Nanjing University of Chinese Medicine, Nanjing, China

**Keywords:** equity, efficiency, productivity, TCM, health resource

## Abstract

**Background:** In this study, we aimed to estimate the equity and efficiency of traditional Chinese medicine (TCM) health resource allocation, utilization, and trend in mainland China from 2013 to 2017.

**Methods:** The data were downloaded from the China Health Statistical Yearbook (2014–2018) and the China Statistical Yearbook (2018). The equity of TCM health resource allocation was evaluated through the Lorenz curve, Gini coefficient (G), and Theil index (T) based on population size and geographical area. The efficiency and productivity of TCM health resource utilization were assessed using the data envelopment analysis-based Malmquist productivity index.

**Results:** TCM health resource had an increasing trend every year. The equity allocated by population (G ranging from 0.1 to 0.3) was better than that by geographic region (G > 0.5). T in the intra-groups was higher than those in the inter-groups. The equity of TCM resource allocation was the middle region > eastern region > western region. Most provinces (29 out of 31) had negative productivity changes, suggesting deterioration in productivity. Moreover, the middle region with higher scale sizes had more redundant inputs than the other two regions. However, the low technological development (all technical values <1) might hinder productive progress.

**Conclusion:** The equity of TCM health allocated by the population was better than that by the geographic region. The intra-regional difference was the main reason for inequity sources. Productivities in more than 97% of provinces are inefficient. The frequency distribution of scale efficiency (score > 1) had increased since 2015. However, the frequency distribution of technical change (score > 1) decreased every year. The slow technological progress and low scale size might be the main reasons for low productivity.

## Introduction

Health resource allocation has been a global issue in the human health service market. The equity and efficiency of the distribution of health resources are regarded as the main goals pursued by public health management and also the basic principles advocated by the World Health Organization (1). Since the 1980s, with the implementation of market-oriented reform in China, the existing health resources and services are struggling to meet the needs of rapid economic development and the aging population (2). The inequity, inefficiency, and limited insurance coverage of health resources have drawn increasing attention from the government. In 2009, the Chinese Ministry of Health launched an ambitious healthcare reform program aiming for universal health coverage for all Chinese citizens by 2020 (3). In the past 10 years, with deepening reforms on health facilities and public hospitals, a zero-price policy of medicine, and full coverage of basic insurance, the ongoing health reform has made great progress (4, 5). The Global Burden of Disease study reported that China's healthcare assessment and the quality index was 77.9 in 2016, which ranked 48 out of 195 countries (6). However, regional disparities still exist in healthcare development (7).

In China, traditional Chinese medicine (TCM) is as equally important as western medicine and plays a vital role in the entire health system (8). As a unique health resource in China, TCM is a part of the traditional culture. TCM services include Chinese herbs, acupuncture, massage, moxibustion, and so on. TCM hospitals are always the main force in medical healthcare and national medical health services. Since 1949, TCM hospitals (including integrated Chinese and western hospitals, and ethnic medicine hospitals) have made great achievements, and the capability of medical services has also constantly improved. However, developing TCM into a viable business is difficult due to the low prices of TCM services. And the fact that equipment and medical facilities are relatively old and slow to update. Hence, the development of new technologies and projects of TCM have been restricted. Moreover, training Chinese medicine professionals is a long process, and introducing high-level talent is difficult. The small proportion of TCM talent in TCM hospitals affects their development. All the results lead to low levels of medical resource utilization, irrational health resource allocation, and low operational efficiency.

Fortunately, along with the promulgation and enforcement of “TCM law” and “the 13th Five-Year Plan of TCM” in 2016, the government has increased its investment in infrastructure construction, deepening the comprehensive reform of TCM hospitals, and training high-level talent. The demand for health services (8), especially TCM services in China with a 1.3 billion population, is increasing. Hence, we aim to use comprehensive indicators including the Lorenz curve, Gini coefficients, Theil index, and data envelopment analysis (DEA)-based Malmquist productivity index (MPI) to explore the changes of the TCM health resource allocation and utilization trend from 2013 to 2017. We aim to provide a reference for government policy decisions based on population and geographical structure.

## Methods

### Data Resources and Region Division

In our study, the data from the China Health Statistical Yearbook (2014–2018) and the China Statistical Yearbook (2018) were collected. The input indicators included health staff (practicing doctors, assistant practicing doctors, registered nurses, pharmacists, and technicians), institutions (TCM hospitals, integrated Chinese and western hospitals, and ethnic medicine hospitals), and bed numbers. On the contrary, the output indicators included outpatient visits and discharged patients from the above institutions. China has 23 provinces, five autonomous regions, four municipalities, and two special administrative areas. In our study, we did not include the data of Hong Kong, Macao, and Taiwan because of the inconsistent statistical standards. The remaining regions are divided into three different regions geographically, that is, the eastern, middle, and western areas. The eastern region contains eight provinces (Hebei, Liaoning, Jiangsu, Zhejiang, Fujian, Shandong, Guangdong, and Hainan) and three municipalities (Beijing, Tianjin, and Shanghai). The middle region includes eight provinces (Shanxi, Jilin, Heilongjiang, Anhui, Jiangxi, Henan, Hubei, and Hunan). Moreover, the western region includes six provinces (Sichuan, Guizhou, Yunnan, Shaanxi, Gansu, and Qinghai) and five autonomous regions (Inner Mongolia, Guangxi, Tibet, Ningxia, and Xinjiang) and one municipality (Chongqing).

### Measuring Tools

The Lorenz curve was first proposed by a famous economist Max Otto Lorenz (9). The Gini coefficient was developed by Corrado Gini based on the Lorenz curve (9). Both were widely used to determine the equality of resource distribution (10–12). The former is a good approach to visually judge the degree of inequality in the population and geographical allocation. The x-axis is the cumulative proportion of population or geography, while the y-axis is the cumulative proportion of three input indicators, respectively. The diagonal line of the square is considered the absolute equality curve.

Given the Gini coefficient, the value (G) is calculated as the areas between the Lorenz curve and the perfect equality curve, compared with the areas under the absolute equality curve. G is between 0 and 1. The closer the value to 0, the more equitable the distribution, and vice versa. We regard G < 0.2 as absolute equality, 0.2 < G < 0.3 as relative equality, 0.3 < G < 0.4 as proper equality, 0.4 < G < 0.5 as relative inequality, and G > 0.5 as serious inequality (13). G is calculated as follows:

(1) $$\begin{array}{l} G=\frac{1}{2}\overset{k}{\underset{i=1}{\sum }}|X_{i}-Y_{i}|, \end{array}$$

where X_i_ represents the relative portions of three input indicators, and Y_i_ represents the relative proportion of population or geography. κ represents the total numbers of regions in China.

The Theil index is applied to measure equality to explain the sources of inequality. The value (T) is between 0 and 1. The closer the value to 0, the greater the equality, and vice versa. The formula (14) of T is given as follows:

(2) $$\begin{array}{l} T=\overset{n}{\underset{i=1}{\sum }}P_{i}log\frac{P_{i}}{Y_{i}}, \end{array}$$

where P_i_ is the proportion of the three different regions, accounting for the total population. Y_i_ is the health resources of the three different regions, accounting for the total health resources.

The Theil index can be divided into an intra-group (T_intra−group_) and an inter-group (T_inter−group_). The formulas are presented as follows:

(3) $$\begin{array}{lll} T_{\mathrm{int}ra} & = & \overset{k}{\underset{g=1}{\sum }}p_{g}t_{g}, \end{array}$$

(4) $$\begin{array}{lll} T_{\mathrm{int}er} & = & \overset{k}{\underset{g=1}{\sum }}p_{g}\ln\;\frac{p_{g}}{y_{g}}, \end{array}$$

where t_g_ means the Theil index of the three regional groups. P_g_ and y_g_ have the same meaning as P_i_ and Y_i_ of equation (2).

The Chinese government has more control over the inputs of TCM services than the outputs of health resources. Hence, an output-oriented DEA-based Banker-Charnes-Cooper model and MPI were then chosen to measure the dynamic efficiency of the TCM service system in our study. DEA is a method to assess the efficiency and productivity of decision-making units (DMUs) by using multiple inputs and outputs, and MPI is used to calculate the total factor productivity changes (Tfpch). The score of Tfpch > 1 means that the total factor productivity has improved, the cost has reduced and productivity has increased. The Tfpch can be divided into technical change (Techch) and technical efficiency change (Effch). The latter can also be decomposed into pure efficiency (Pech) and scale efficiency (Sech) (15). Techch is applied to assess the impact of technological advances on product changes. The score of Techch > 1 means that the cost economy and productivity are improved by technological innovation. Then, Effch is used to measure the innovation degree of technical progress within two periods. The score of Effch > 1 means that the improvement is efficient. Pech reflects the managerial efficiency, whereas Sech mainly reflects the scale changes of different DMUs (16). The score of Pech < 1 reflects the need of management enhancement. The score of Sech < 1 means that there is an urgent need for scale adjustment. All these data were used by the DEAP V.2.1 version (17).

## Results

### Equity of Health Resource Allocation

Table 1 shows the changes in health resource allocation per 1,000 persons and 1,000 m^2^. Health resources had an increasing trend from 2013 to 2017. The average annual growth rates of institutions, beds, and health staff in 2013 to 2017 were 13.69, 19.58, and 15.31%, respectively.

**Table 1:** Health resource allocation trend from 2013 to 2017.

**Year**	**Institutions**			**Beds**			**Health staff**		
	**/1,000 persons**	**/1,000 m^**2**^**	**Total**	**/1,000 persons**	**/1,000 m^**2**^**	**Total**	**/1,000 persons**	**/1,000 m^**2**^**	**Total**
2013	0.0304	0.0436	41,966	0.5836	0.8342	794,160	0.4403	0.6217	599,114
2014	0.0319	0.0453	43,635	0.6414	0.9104	877,255	0.4724	0.6706	646,152
2015	0.0339	0.0483	46,541	0.6966	0.9937	957,523	0.5055	0.7211	694,827
2016	0.0358	0.0514	49,527	0.7475	1.0726	1,033,547	0.5393	0.7739	745,725
2017	0.0390	0.0563	54,243	0.8170	1.1785	1,135,615	0.5731	0.8268	796,704

**Figure 1:**
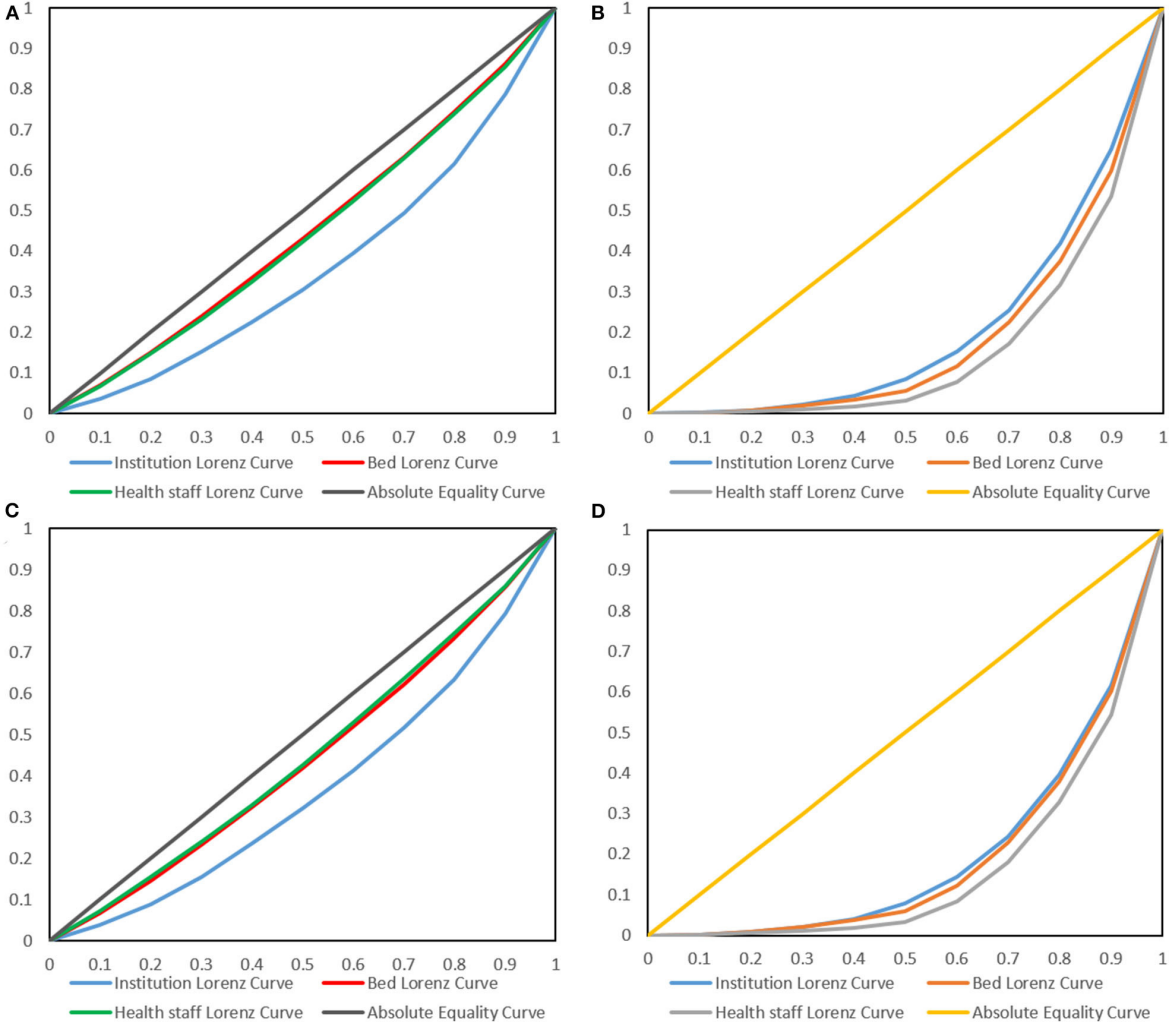
Lorenz curves of health resources in 2013 **(A,B)** and 2017 **(C,D)**. **(A,C)** are the Lorenz curves allocated by population, whereas **(B,C)** are the Lorenz curves allocated by geographical regions.

The Lorenz curves in terms of population size and geographical area from 2013 to 2017 were all drawn. However, we only showed the 2013 and 2017 Lorenz curves (Figure 1) because the others had the same trends. From the Lorenz curves (Figures 1A,C), the beds and health staff curves were closer to the absolute equality curve, whereas the institution's curves were farther away from the absolute equality curve. The results indicated that the beds and health staff were more equitable than the institutions allocated by the population size. In the Lorenz curves (Figures 1B,D), we found that the institutions and beds curves were closer to the absolute equality curve, whereas the health staff curves were farther from the absolute equality curve. These results suggested that the institutions and beds were more equitable than the health staff allocated by the geographical region.

Given the Gini index from Table 2, only the G of beds and health staff allocated by population size was <0.2. This result suggested that they were absolutely equitable. The G of institutions in terms of the population was between 0.2 and 0.3, showing a decreasing trend. This result indicated that the equity of the institution allocation was improving each year. However, the G allocated by geographical regions were all more than 0.5, which indicated that their equity was worse. The main reason might be the regional disparities in different regions (eastern, middle, and western). The average number of institutions in the eastern, middle, and western regions was 0.1536, 0.072, and 0.027, respectively. The average annual growth rates were 9.47, 5.29, and 4.99%, respectively. As for beds, the average numbers in the three regions were 3.3016, 1.7524, and 0.4465, respectively. Moreover, the average annual growth rates were 8.46, 9.25, and 10.52%, respectively. With regard to health staff, the average numbers of the three regions were 2.7533, 1.3081, and 0.2589, and the average annual growth rates were 6.41, 6.87, and 9.72%, respectively.

**Table 2:** Gini index of health resource by population and geographical region trend from 2013 to 2017.

**Year**	**Allocation by population**			**Allocation by geographical region**		
	**Institutions**	**Beds**	**Health staff**	**Institutions**	**Beds**	**Health staff**
2013	0.2855	0.1020	0.1080	0.5888	0.6199	0.6751
2014	0.2793	0.1074	0.1090	0.5821	0.6177	0.6738
2015	0.2798	0.1089	0.1080	0.5866	0.6175	0.6710
2016	0.2754	0.1130	0.1028	0.5905	0.6181	0.6661
2017	0.2644	0.1166	0.1024	0.5970	0.6156	0.6671

The intra- and inter- groups of the Theil index were used to further explore the sources of the inequity (Table 3). The results suggested that the intra-regional differences were the main reasons for resource inequity. We further found that these differences were mainly due to intra-region disparities. The contribution rate of intra-health sources (institutions, beds, and health staff) accounted for 99.8, 94, and 83.5%, respectively. Table 4 shows T of every region trend from 2013 to 2017. We found that T of all health resources were the smallest in the middle region and the largest in the western region. This result suggested that the middle region had the most equitable health resource distribution, whereas the western region had the worst equitable allocation. All results indicated that the inequity of TCM resources mainly came from the intra-western region.

**Table 3:** Theil index of health resource trend from 2013 to 2017.

**Items**	**2013**	**2014**	**2015**	**2016**	**2017**
**INSTITUTIONS**					
T	0.2250	0.2229	0.2266	0.2287	0.2341
T_Intra−groups_	0.2241	0.2226	0.2266	0.2287	0.2336
T_Inter−groups_	0.0009	0.0003	0.0001	0.000	0.0006
**BEDS**					
T	0.1896	0.1909	0.1897	0.1922	0.1922
T_Intra−groups_	0.1763	0.1791	0.1779	0.1812	0.1823
T_Inter−groups_	0.0134	0.0117	0.0117	0.0109	0.0099
**HEALTH STAFF**					
T	0.2386	0.2370	0.2313	0.2278	0.2311
T_Intra−groups_	0.1923	0.1961	0.1933	0.1932	0.1982
T_Inter−groups_	0.0462	0.0409	0.0381	0.0346	0.0329
**OUTPATIENT VISITS**					
T	0.2887	0.2911	0.2891	0.2924	0.2912
T_Intra−groups_	0.2082	0.2083	0.2082	0.2120	0.2157
T_Inter−groups_	0.0805	0.0828	0.0808	0.0804	0.0756
**DISCHARGED PATIENTS**					
T	0.2344	0.2307	0.2319	0.2542	0.2299
T_Intra−groups_	0.2222	0.2196	0.2217	0.2441	0.2224
T_Inter−groups_	0.0122	0.0111	0.0101	0.0101	0.0075

**Table 4:** Theil index of the trend of health resources among different regions from 2013 to 2017.

**Items**	**2013**	**2014**	**2015**	**2016**	**2017**
**INSTITUTIONS**					
Eastern	0.2117	0.2153	0.2153	0.2357	0.2597
Middle	0.0621	0.0588	0.0646	0.0632	0.0568
Western	0.3405	0.3372	0.3341	0.3301	0.3248
**BEDS**					
Eastern	0.1940	0.1966	0.1972	0.2028	0.2086
Middle	0.0859	0.0931	0.0964	0.0973	0.0969
Western	0.2440	0.2421	0.2346	0.2372	0.2333
**HEALTH STAFF**					
Eastern	0.2062	0.2066	0.1996	0.2104	0.2157
Middle	0.0920	0.0985	0.1030	0.0996	0.1034
Western	0.2992	0.3012	0.2937	0.2783	0.2827
**OUTPATIENT VISITS**					
Eastern	0.1972	0.1969	0.1967	0.2016	0.2074
Middle	0.0814	0.0865	0.0913	0.0981	0.1007
Western	0.3320	0.3319	0.3251	0.3321	0.3216
**DISCHARGED PATIENTS**					
Eastern	0.2391	0.2400	0.2410	0.3192	0.2459
Middle	0.1362	0.1384	0.1421	0.1374	0.1399
Western	0.2870	0.2748	0.2759	0.2699	0.2721

### Efficiency of Health Resource Allocation

From the descriptive statistics trend from 2013 to 2017 (Table 5), we found that both input and output indicators increased annually. The data from 2017 were applied to show the slacks of inputs and outputs (Table 6). The inputs and outputs in the inefficient provinces need to adjust their variations. Approximately 19 provinces, two autonomous regions, and two municipalities had decreasing returns to scale. Amongst them, four provinces should reduce three inputs. Seven provinces and one autonomous region should adjust institutions and bed inputs. Two provinces should reduce institutions and health staff, whereas two provinces should adjust both beds and health staff. Four provinces, one autonomous region, and one municipality should adjust their health staff. Moreover, Tianjin and Hebei should not only adjust the inputs but also improve the outputs (Table 6).

**Table 5:** Descriptive statistics trend of inputs and outputs from 2013 to 2017.

**Year**	**Items**	**Inputs**			**Outputs**	
		**Institutions**	**Beds**	**Health staff**	**Outpatient visits**	**Discharged patients**
2013	Max	5,022	59,477	48,249	8,948	1,856,584
	Min	100	1,222	1,346	144	20,765
	Mean	1,354	25,618	19,370	2,613	734,189
2014	Max	5,077	67,265	50,382	9,625	2,049,837
	Min	102	1,489	1,231	171	29,060
	Mean	1,408	28,300	20,883	2,820	818,431
2015	Max	5,280	71,649	53,855	9,791	2,179,909
	Min	115	1,941	1,437	217	33,147
	Mean	1,501	30,888	22,460	2,932	868,214
2016	Max	5,536	77,824	57,168	10,750	2,367,495
	Min	117	1,879	1,546	217	34,610
	Mean	1,598	33,340	24,106	3,104	910,393
2017	Max	5,931	86,064	62,465	11,357	2,719,521
	Min	149	2,193	1,872	249	37,670
	Mean	1,750	36,633	25,761	3,286	1,061,601

**Table 6:** Slacks of inputs and outputs in 2017.

**Provinces**	**Inputs**			**Outputs**	
	**Institutions**	**Beds**	**Health staff**	**Outpatient visits**	**Discharged patients**
Beijing	0	0	0	0	0
Tianjin	0	−2948.680	0	14.631	0
Hebei	−243,094.171	0	−485.968	6.912	0
Shanxi	0	0	−1,552.857	0	0
Inner Mongolia	0	0	−1,578.506	0	0
Liaoning	0	0	−882.423	0	0
Jilin	0	0	−710.180	0	0
Heilongjiang	0	−608.632	−331.049	0	0
Shanghai	0	0	0	0	0
Jiangsu	−624,295.457	−13,344.929	0	0	0
Zhejiang	0	0	0	0	0
Anhui	−528,760.717	−12,417.236	0	0	0
Fujian	0	0	−160.221	0	0
Jiangxi	−226,022.560	−3,264.524	0	0	0
Shandong	−608,706.100	−17,189.485	−17.937	0	0
Henan	−758,414.024	−21,243.353	0	0	0
Hubei	−536,501.730	−5,417.382	0	0	0
Hunan	−762,269.571	−19,295.378	−412.403	0	0
Guangdong	0	0	0	0	0
Guangxi	−172,828.226	−5,120.625	0	0	0
Hainan	0	−680.028	−9.715	0	0
Chongqing	0	0	−813.431	0	0
Sichuan	−814,776.018	−4,647.753	−3,037.551	0	0
Guizhou	−222,670.741	−5,438.150	0	0	0
Yunnan	−125,135.601	−1,868.266	0	0	0
Tibet	0	0	0	0	0
Shaanxi	−78,967.758	−11,919.417	−166.413	0	0
Gansu	−27,777.106	0	−440.365	0	0
Qinghai	0	0	0	0	0
Ningxia	0	0	0	0	0
Xinjiang	0	0	0	0	0

### Productivity of Health Resource Allocation

The productivity of health resource allocation was measured by the MPI, as shown in Table 7. The annual mean of Tfpch was 1.006, which showed an increasing trend of 0.6% from 2013 to 2017. The increase in Tfpch was mainly due to a 0.6% increase in Techch, even though the Techch in 2014–2015 was down by 1.5%. Moreover, we calculated the MPI in different provinces, autonomous regions, and municipalities, as presented in Table 8. We found that only Fujian and Henan went through positive productivity changes (the scores of Tfpch > 1) from 2013 to 2017. However, the others had negative productivity changes, which suggested a deterioration in productivity. Furthermore, the scores of Techch in all provinces, autonomous regions, and municipalities were < 1, which indicated that their technical efficiency was inefficient.

**Table 7:** MPI and frequency distribution of health resource allocation by year.

**Year**	**Effct**	**Techch**	**Pech**	**Sech**	**Tfpch**
2013–2014	1.000	1.012	1.000	1.000	1.012
2014–2015	1.000	0.985	1.000	1.000	0.985
2015–2016	1.000	1.020	1.000	1.000	1.020
2016–2017	1.000	1.007	1.000	1.000	1.007
Mean	1.000	1.006	1.000	1.000	1.006
**Frequency distribution (2013–2014)**					
>1	13	10	13	10	9
1	4	1	9	4	0
<1	14	20	9	17	22
**Frequency distribution (2014–2015)**					
>1	12	4	16	10	5
1	3	0	8	3	0
<1	16	27	7	18	26
**Frequency distribution (2015–2016)**					
>1	16	1	8	18	4
1	4	0	10	3	0
<1	11	30	13	10	27
**Frequency distribution (2016–2017)**					
>1	21	0	17	20	6
1	3	0	8	3	0
<1	7	31	6	8	25

**Table 8:** MPI of health resource allocation in each province.

**Provinces**	**Effct**	**Techch**	**Pech**	**Sech**	**Tfpch**
Beijing	1.000	0.942	1.000	1.000	0.942
Tianjin	0.999	0.956	1.001	0.998	0.954
Hebei	1.036	0.945	1.023	1.013	0.979
Shanxi	1.024	0.953	1.013	1.010	0.975
Inner Mongolia	0.989	0.967	1.005	0.984	0.957
Liaoning	0.997	0.955	0.989	1.007	0.952
Jilin	1.013	0.951	1.002	1.010	0.964
Heilongjiang	0.987	0.953	0.982	1.005	0.941
Shanghai	1.000	0.982	1.000	1.000	0.982
Jiangsu	1.019	0.976	1.018	1.001	0.995
Zhejiang	1.032	0.954	1.000	1.032	0.985
Anhui	0.951	0.973	1.005	0.947	0.926
Fujian	1.057	0.956	1.007	1.050	1.010
Jiangxi	1.016	0.961	1.012	1.003	0.976
Shandong	1.040	0.957	1.006	1.034	0.995
Henan	1.027	0.981	1.034	0.994	1.008
Hubei	1.008	0.977	1.027	0.982	0.985
Hunan	1.003	0.971	0.998	1.005	0.974
Guangdong	1.018	0.954	1.000	1.018	0.971
Guangxi	1.011	0.961	1.005	1.006	0.972
Hainan	1.071	0.928	1.007	1.063	0.993
Chongqing	0.930	0.989	0.989	0.940	0.920
Sichuan	0.977	0.998	1.000	0.977	0.975
Guizhou	0.991	0.978	1.002	0.989	0.969
Yunnan	0.944	0.988	1.003	0.941	0.933
Tibet	1.000	0.908	1.000	1.000	0.908
Shaanxi	1.001	0.954	1.000	1.000	0.955
Gansu	0.948	0.984	0.998	0.950	0.933
Qinghai	1.018	0.953	1.000	1.018	0.971
Ningxia	0.974	0.955	1.000	0.974	0.929
Xinjiang	0.939	0.973	1.000	0.939	0.914

## Discussion

Along with the implementation of the medical reform plan in 2009, medical service allocation has undergone great changes, specifically the outline of the strategic program for the development of TCM by the State Council in 2016. TCM has entered a period of fast development and has set several nationwide goals to be accessed by the end of 2020. One of the tasks is to build a nationwide medical service network to ensure that all citizens can enjoy the basic services of TCM. As TCM has achieved long-term development, some equity and efficiency issues still need to be explored. We aimed to help the Chinese government to optimize TCM resource allocation.

Currently, we used comprehensive economic methods to reveal the extent, nature, and source of TCM health resource allocation in mainland China from 2013 to 2017. We found the overall numbers of TCM institutions and beds and health staff, and the numbers per 1,000 persons and 1,000 m^2^ grew steadily. This is indeed the result of the government's emphasis on health resources over the years. The equity of health resource allocation was measured using the Gini and Theil indexes. The former was applied to judge the overall inequity, and the latter was used to find the source of inequity. Beds, health staff, and institutions are distributed fairly in terms of population. G in terms of population ranged from 0.10 to 0.29. The equity of TCM resources in terms of population was health staff > beds > institutions. However, the results of the Gini coefficient based on geographical size were not ideal. G of the geographical areas ranged from 0.58 to 0.68, and the scores were all above 0.5, which suggested great regional disparities. The findings were consistent with those of a previous study, which indicated the remaining regional disparities, especially between the eastern and western regions (7). The equity of TCM resource by the geographical region was institutions > beds > health staff. The results were also in line with those of a published study (18). Similarly, evidence indicated that the equity of health resource allocation in terms of population size was more equitable than that in terms of the geographic region (11, 13). From the health allocation documents issued by the Chinese government, it is not hard to ascertain that some documents were based on population allocation rather than geographical area distribution. This also explains why it is a more equitable allocation by population size than by geographical structure.

Theil analysis was efficient in finding the sources of inequality, which consisted of intra-group and inter-group inequality and the contribution rate within and between groups (19, 20). After the analysis, we found that the intra-group difference was the main reason for the inequity of TCM health resource allocation. The equity in three of the regions was health staff > beds > institutions, which was consistent with the Gini coefficient by population. Moreover, the inequity by geographic region was western > eastern > middle. From the number of institutions in the western areas, we also found that Sichuan had 50 times that of Tibet, and 8 out of 12 provinces in 2017 had the low average number of institutions. This finding might explain the large gap in the western areas. Given national conditions, the imbalance of economic development of the western region is due to the misdistribution of health allocation regardless of the downtrend in the annual contribution rate. Surprisingly, the contribution rates of health resources in the eastern and middle regions have been steadily growing. The inequality gap between the western and middle areas are decreasing.

From the efficiency and productivity analysis, we found that all provinces in the middle region had redundant inputs, which had not been entirely utilized. However, the inputs in the western region with limited technical levels were better utilized than those in the middle area. Four out of 12 provinces (Qinghai, Ningxia, Tibet, and Xingjiang) in the western region had no adjustment in inputs. Moreover, we found that Beijing (eastern), Shanghai (eastern), and Tibet (western) should not need to adjust their inputs, outputs, and scale under the present technical level. Zhejiang (eastern), Guangdong (eastern), and Qinghai (western) should reduce their scale, and Ningxia (western) and Xinjiang (western) also need to adjust their scale. Productivity had negative changes between 2014 and 2015, whereas the other periods had positive changes. We also found that the frequency distribution of Sech (score > 1) had increased since 2015. However, the frequency distribution of Techch (score > 1) decreased annually. And the scores of Techch in all provinces, autonomous regions, and municipalities (Table 8) were <1. It showed a low level of technical development. Technical improvement plays a pivotal role in the short-term development of medical service resources (16). As for Tfpch, it can be decomposed into Effct and Techch, whereas Effct can be divided into Pech and Sech, that is, Tfpch = Effct ^*^ Techch; Effct = Pech ^*^ Sech (17). The scores of Sech in 12 out of 31 provinces, autonomous regions, and municipalities were <1. It might be the main reason causing the inefficient improvement of production in these provinces, autonomous regions, and municipalities. Hence, we should improve technological levels and optimize scale continuously to obtain long-term development.

Our study has some limitations. Firstly, given the medical environment in China, many other indicators, such as the workload of health staff, turnover rate of beds, and hospital expenses were not included in our analysis. Our findings might not reflect the entire status of TCM resources in China. Secondly, the health resource density index (HRDI) can mediate the influence of demographic or geographical dimensions to reduce the bias resulting from a single aspect of population or geographical region. HRDI by the population size or geographic area was not measured in our study, which might cause some bias in our results. Thirdly, the efficiency and productivity values were not adjusted because of the limitation of the DEA method (21). Fourthly, the TCM data after 2017 were not available in the China Health Statistical Yearbook. Hence, we chose the recent 5-year period. Future studies could employ additional indicators, longer periods, and better analysis approaches.

## Conclusion

In our study, we comprehensively evaluated the equity and efficiency of the TCM resource trend for over 5 years. We found that TCM resources became increasingly equitable in recent years. The equity in terms of the population size was higher than that by geographical factor. The middle area was more equitable than the western area, which was consistent with the results of the DEA analysis. Productivity in most provinces had negative changes. The middle region with large scales had redundant inputs. However, the western region with low scale size had few redundancies. Although the increasing inputs shortened the regional gaps and improved equity, slow technology development still hindered productive progress. Herein, with the existing resource being allocated rationally and used effectively, technological progress and scale levels should be improved.

## Data Availability Statement

The datasets presented in this study can be found in online repositories. The names of the repository/repositories and accession number(s) can be found below: China Health Statistical Yearbook (2014–2018) and the China Statistical Yearbook (2018).

## Author Contributions

ZL conceived and designed the study. LY collected the data. YB and ST provided several suggestions for manuscript revision. All authors contributed to the article and approved the submitted version.

## Conflict of Interest

The authors declare that the research was conducted in the absence of any commercial or financial relationships that could be construed as a potential conflict of interest
